# Bacterial Contamination of Children’s Toys in Rural Day Care Centres and Households in South Africa

**DOI:** 10.3390/ijerph16162900

**Published:** 2019-08-13

**Authors:** Solanka Ellen Ledwaba, Piet Becker, Afsatou Traore-Hoffman, Natasha Potgieter

**Affiliations:** 1 Department of Microbiology, University of Venda, Thohoyandou 0950, South Africa; 2 Research Office, Faculty of Health Sciences, University of Pretoria, Pretoria 0028, South Africa

**Keywords:** diarrhoeagenic *Escherichia coli*, rural, toys, WASH conditions

## Abstract

Background: Young children exhibit a high susceptibility to several diarrhoea-causing bacterial microorganisms. In this study, the prevalence of fecal contamination on children’s toys was determined using total coliform and *E. coli* as bacterial fecal indicators. The prevalence of diarrhoeagenic *E. coli* strains were used as an indication of the potential health risks. Materials and Methods: A cross-sectional descriptive study was carried out for 3 months in rural communities in the Vhembe district, Limpopo province of South Africa. Nonporous plastic toys (n = 137) used by children under 5 years of age in households and day care centres (DCCs) from rural villages were collected for assessment. New toys (n = 109) were provided to the households and DCCs and collected again after 4 weeks. Microbiological assessment was carried out using the Colilert^®^ Quanti-Tray/2000 system. Diarrhoeagenic *E. coli* strains were identified using a published multiplex PCR protocol. Results: Water, sanitation and hygiene (WASH) conditions of the children in the households and DCCs were assessed. Statistical analysis was used to identify the relationship between fecal contamination of the existing and introduced toys. All the existing and introduced toy samples, both from DCCs and households, tested positive for total coliform counts and 61 existing and introduced toy samples tested positive for *E. coli* counts. Diarrhoeagenic *E. coli* strains identified included EHEC, ETEC, EPEC, EIEC and EAEC. Conclusions: The results indicated that water, sanitation and hygiene conditions could be responsible in the contamination of children’s toys and the transmission of diarrhoea to young children.

## 1. Introduction

Diarrhoea claims almost 500,000 lives yearly in children under the age of 5 years [1]. In addition to the high number of deaths and the effects of diarrhoea in children that survive, many of these children still have recurrent diarrhoea [2] In developing countries diarrhoea is the second leading cause of death among children less than five years of age [3]. Water, sanitation and hygiene (WASH) are important as these aspects act as a link to faecal–oral disease transmission in young children [4].

Several studies globally have shown that diarrhoea-causing organisms are prevalent on fomites. Young children share their toys, play on dirty floors and put items in their mouths which all could contribute to the transmission of diarrhoea-causing pathogens [5,6,7,8,9,10,11,12,13,14,15,16,17,18,19]. In Atlanta (USA) toys tested from DCCs were contaminated with faecal coliforms [6]. In Mauritius [13] and New Zealand [9], it was found that soft toys tested from DCCs, households and waiting rooms had higher levels of bacterial contamination compared to nonporous toys. In Costa Rica, it was reported that paediatric hospital toys were contaminated with *Staphylococcus* spp., *Bacillus* spp., *Pseudomonas* spp., *Sternotrophomonas malthophilia* and *Enterococcus* spp. and the toys were associated with nosocomial infections in children [10]. Stauber and colleagues (2013) studied toy contamination and the association with water, sanitation and hygiene (WASH) conditions in Honduras (USA) and found that toys from rural households were contaminated with total coliform and *E. coli,* and WASH conditions were found to play an important role in the faecal oral spread of diarrhoeal bacteria [16]. 

The majority of people living in the rural communities in South Africa in Limpopo province in the Vhembe district still lack access to improved water, sanitation and hygiene conditions [20,21]. Most of the households use open water sources (such as rivers) which are shared by humans and animals and the water is often not treated. Some people still lack adequate sanitary facilities for disposal of human excreta and children’s diapers. Children living in Vhembe district have been previously reported to be infected with diarrheal pathogens such as diarrhoeagenic *E. coli* [22,23], *Shigella spp.*, *Salmonella spp.* [23], rotavirus [23,24] and norovirus [23,25]. Diarrheagenic *E. coli* strains are perceived to be dangerous as they can result in gastrointestinal, urinary and central nervous systems diseases in humans [26].

Very little is known on the role that children’s toys from households and DCCs in rural areas in Africa play in the transmission pathway of diarrhoea-causing organisms in children under the age of five years. No study was found in South Africa on contamination of children’s toys in rural households or DCCs. This study was therefore aimed at determining bacterial contamination using total coliforms and *E. coli* as indicators of contamination on children’s toys in rural communities of the Vhembe district.

## 2. Materials and Methods

### 2.1. Ethical Consideration

The project was registered at the University of Venda and ethical clearance was obtained from the ethical board (SMNS/13/MBY/08). The primary caretaker (parent) in each household and the owner of each DCC was approached and each provided with a consent form to sign. A questionnaire was used to collect demographic data on water, sanitation and household/DCC profiles. 

### 2.2. Study Site

A cross-sectional study was carried out for 3 months (from September to November) during spring (dry season) in the rural communities of the Vhembe region of the Limpopo province of South Africa. Rural households from Tshikonelo and Mavhunga villages (n = 64) and DCCs (n = 6) from the Mavhunga, Tshikonelo and Maugani villages were randomly recruited in the Vhembe region to be part in this study. The DCCs had about two to four staff members and the number of children being cared for, ranged between 10 and 30 children per DCC.

### 2.3. Collection of Toys

Plastic toy samples were purchased from a wholesale outlet in Johannesburg, South Africa. All toys were designed for small children. The study concentrated on plastic toys because they are nonporous and easy to clean.

**Figure 1:**
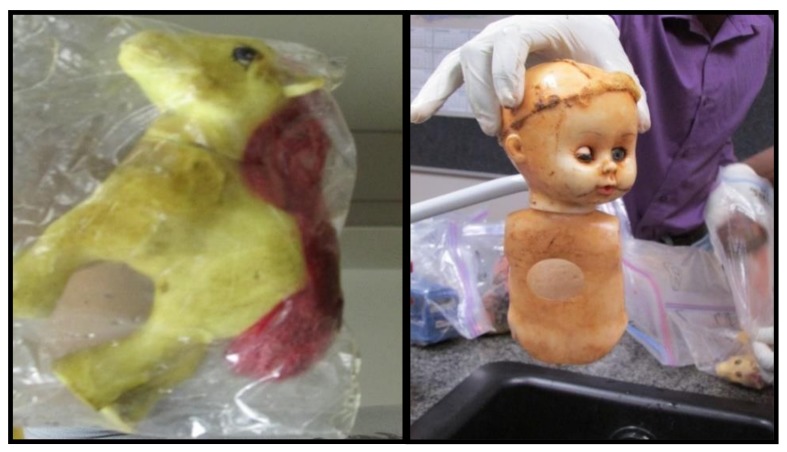
Existing toys.

**Figure 2:**
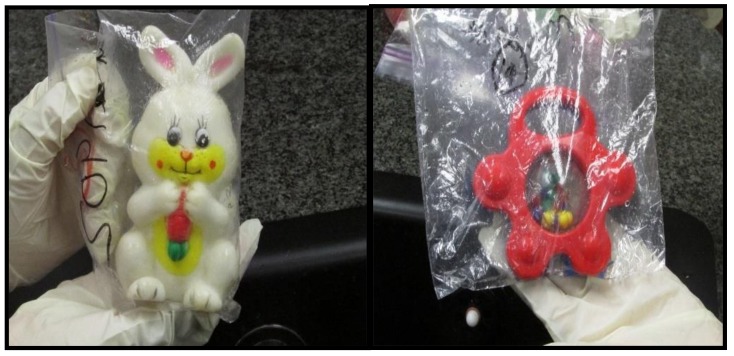
Introduced toys.

In the rural villages of Tshikonelo and Mavhunga, one nonporous toy that was used by the youngest child under five years of age in each of the study households were first collected in a sterile collection bag containing 110 mL phosphate-buffered saline (PBS) (pH 7.4) and kept on ice in a cooler box. A total of 54 toys were collected and this toy was labelled the existing toy (Figure 1). Each of the households were then provided with a new toy (labelled the introduced toy) to play with for 4 weeks. After 4 weeks, the existing toy was returned and the introduced toy (Figure 2) was collected in a sterile collection bag containing 110 mL PBS (pH 7.4) and kept on ice in a cooler box for assessment. An additional ten households of which had a child less than 5 years and did not have any toy in each village were randomly selected to be part of the study. New toys were provided to each of the households for the child to play with and collected after 4 weeks in a sterile collection bag containing 110 mL PBS (pH 7.4) and kept on ice in a cooler box for assessment.

From the six DCCs, only nonporous toys (n = 83) were each collected in a separate sterile collection bag containing 110 mL PBS (pH 7.4) and kept on ice in a cooler box for assessment. Only four of these DCCs were provided with new toys after two DCCs withdrew from the study. Four weeks later, the introduced toys were collected in a separate sterile collection bag each containing 110 mL PBS (pH 7.4) and kept on ice in a cooler box for assessment.

All samples from households and DCCs were assessed within 2 h from collection at the Microbiology laboratory of the University of Venda in the Vhembe district of the Limpopo Province.

### 2.4. Microbiological Assessment of Toy Samples

In the laboratory, each toy was massaged for approximately 2 min in the ziplock bag containing the sterile PBS in order to loosen bacteria on each toy. A total of 100 mL of the PBS solution was then removed aseptically from each bag and tested using the Colilert^®^ Quanti-Tray/2000 system according to the manufacturer’s instructions. The Quanti-Trays were incubated for 18 h at 35 °C. After incubation, the Quanti-Trays/2000 were examined under long wave (360 nm) ultraviolet light, and wells that turned yellow were counted as total coliforms, while wells that turned yellow and fluorescent were counted as *E. coli* positive (IDEXX).

### 2.5. Molecular Characterisation of Diarrhoeagenic E. coli Strains

The multiplex PCR (mPCR) protocol published by Omar and Barnard was used to detect the presence of diarrhoeagenic *E. coli* genes in all samples [27]. Briefly: A total of 2 mL of the positive sample were removed from the ten positive randomly selected *E. coli* wells of the Colilert Quanti-Tray/2000 into sterile 2 mL Eppendorf tubes. The tubes were centrifuged for 10 min at 70 °C and 250 µL of 100% ethanol with L6 lysis buffer (Seven Biotech, Kidderminster, UK) were added to increase binding of DNA and incubated for 10 min at 56 °C. Celite (50 µL) was added and incubated for 10 min at room temperature. After incubation, 400 µL of the solution was loaded to a spin column and centrifuged for 30 s at 13,000 rpm. The solution was centrifuged for 30 s at 13,000 rpm and the pallet was washed with 400 µL L2 wash buffer (Seven Biotech) and centrifuged for 30 s at 13,000 rpm. Ethanol (400 µL of 70%) was added to the pellet and centrifuged for 30 s at 13,000 rpm. About 100 µL AE buffer (Qiagen, Germantown, MD, USA) was added to the pellet and incubated for 2 min at 56 °C, centrifuged for 2 min at 13,000 rpm. DNA was stored at −20 °C.

All m-PCR reactions were performed in a Biorad Mycycler^TM^ Thermal cycler in a total volume of 20 µL. The primers used are shown in Table 1. Each reaction consisted of Qiagen^®^ PCR multiplex mix (HotstartTaq^®^ DNA polymerase, m-PCR buffer and deoxy Nucleotide Triphosphate (dNTP) mix (Qiagen); 2 µL of the primer mixture (0.1 LM of *mdh* and *lt* primers, 0.2 LM of *ial*, *eagg*, *astA*, *bfp* and *gapdh* primers, 0.3 LM of eaeA and *stx2* primers, 0.5 LM of *stx1* and *stx2* primers); 2 µL of DNA sample; 1 µL of *gapdh* cDNA and 5 µL of PCR-grade water. The reactions were subjected to an initial activation step at 95 °C for 15 min, 35 cycles that consisted of denaturation at 95 °C for 45 s, annealing at 55 °C for 45 s, extension at 68 °C for 2 min and final elongation at 72 °C for 5 min.

Bacterial DNA was analysed using 2.5% (*w*/*v*) agarose gel in TAE buffer (40 mmol^−1^ Tris acetate; 2 mmol^−1^ Ethylene-Diamine-Tetra-Acetic (EDTA, pH 8.3) with 0.5 µgmL^−1^ Ethiduim Bromide). DNA was electrophoresed for 1–2 h in electric field strength of 8 V·cm^−1^ gel. The DNA was then visualised using UV light (Gene Genius Bio Imaging system, Vacutec^®^, Costa Mesa, CA, USA). The relative sizes of the DNA fragments were estimated by comparing their electrophoretic mobility with that of the standards run with the samples on each gel, either 1 kB or 100 bp markers (Thermofisher Scientific, Waltham MA, USA).

### 2.6. Statistical Analysis

Bacterial concentration data from toy samples and data from questionnaires (age, sex, water, sanitation and hygiene conditions of the participants) were entered into Microsoft Excel spread sheets. The concentration data for the whole range of toy samples analysed for faecal bacteria were included, 0.5 MPN/100 mL was assigned to samples that fell below the lower detection limit of <1 MPN/100 mL, while 2420 MPN/100 mL was assigned for values that were above the detection limit of 2419.6 MPN/100 mL. Descriptive analytical frequencies, percentages and 95% confidence intervals (CI) were used. Counts were summarised using geometric means and 95% CI for toys that tested positive. McNemar’s test for symmetry was employed to assess shift away from agreement between existing and introduced toys.

## 3. Results

### 3.1. Descriptive Analysis of Questionnaires from Households and Day Care Centres

Sanitation demographics of the households and the DCCs are presented in Table 2. The majority of households had access to sanitary facilities ranging from pit latrines (83%) to the bucket system (9%). The children defecated in open spaces in the yard (30%), in the bush (9%) or used a children’s toilet (35%). Although all the DCCs had toilet facilities, 50% reported that young children still defecated in open spaces in the yard. 

Water demographic data collected from the households and the DCCs is shown in Table 3. In this study, households used protected water sources such as communal taps (41%), tanks (15%) and boreholes (6%) and unprotected water sources such as river water (19%). The DCCs used protected water sources from communal taps (33%) and boreholes (17%). Both households (100%) and DCCs (67%) reported that water is not always available, and they have to use alternative sources for periods ranging from weeks to months. Both the households and DCCs reported that they sometimes have to travel between 50 and 200 m to collect water and transport it back either carrying it on their heads or using a wheelbarrow. 

Hygiene demographics of the households and the DCCs are shown in Table 4. None of the DCCs had a handwashing station close to the toilet, while only 22% of the households had a handwashing station close to the toilet. Approximately 28% of households and 67% of DCCs reported to be using a washing container for hand wash activities. Most of the study households reported to wash hands only after using the toilet (81%), before preparing food (80%) and before cooking meals (80%). All the DCCs reported to wash hands after changing nappies (100%), before preparing food (100%) and before meals (100%). 

### 3.2. Frequencies of Total Coliforms and E. coli on Toys

Table 5 provides an overview of the number of toys (existing and introduced) that were collected during this study for assessment. A total of 246 toy samples were collected and processed for total coliform bacteria and *E. coli* bacteria prevalence. Of these, 137 were existing toys consisting of 54 toys from households and 83 toys from DCCs. A total of 109 new toys were provided (introduced) to both the households and the DCCs for a couple of weeks and then collected. These included 54 toys from the households and 55 toys from the DCCs.

Although 64 new (introduced) toys were given to the households, only 54 toys were finally collected because participants were not at home at the time of collection and some of the participants lost the new toy. Initially, a total of eighty-three (n = 83) existing toys were collected from the six DCCs and assessed. However, two of the DCCs withdrew from the study and a total of 55 new toys were then given to the remaining four DCCs for children to use for 4 weeks, at which time, the existing toys were returned to the DCCs and the introduced toys were then taken for assessment. The geometric mean counts of total coliform bacteria and *E. coli* bacteria on existing and introduced toys are summarised in Table 6. 

Contamination of old vs. new toys within households was assessed using McNemar’s test for symmetry to determine whether discordance was random or not. In the rural households, the McNemar’s test for symmetry showed that there was no statistical difference for total coliform bacterial counts (*p* = 0.8172) and for *E. coli* bacterial counts (*p* = 0.1019) between the existing and the introduced toys. 

### 3.3. Prevalence of Diarrhoeagenic E. coli Strains on Toys

**Figure 3:**
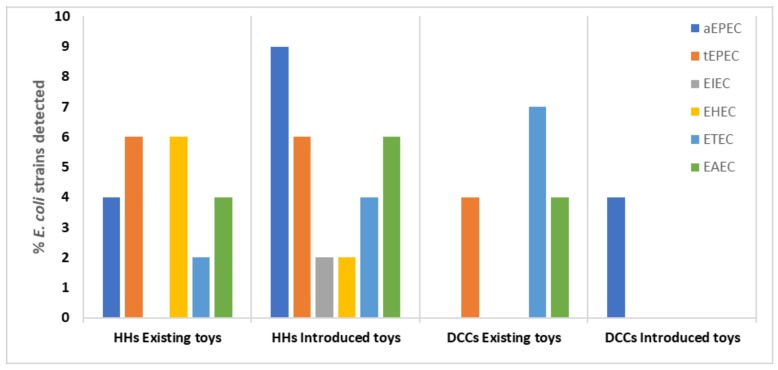
Prevalence of pathogenic *E. coli* strains found on existing and introduced toys (Excluding data from 2 DCCs who withdrew from study; Keywords: EAEC = enteroaggregative *E. coli*, aEPEC = atypical enteropathogenic *E. coli*, tEPEC = typical enteropathogenic *E. coli*, EHEC = enterohemorrhagic *E. coli*, EIEC = enteroinvasive *E. coli*).

Different diarrheagenic *E. coli* were identified from both existing and introduced toys in rural households and DCCs (Figure 3). All *E. coli* samples were confirmed using the *Mdh* primers. No EIEC or EHEC strains were found on existing or introduced toys from the DCCs. Similarly, no EHEC, EAEC or tEPEC strains were detected on the introduced toys in the DCCs, as DCCs did report washing the toys and hands of children. All the diarrhoeagenic *E. coli* strains were however detected in the household toy samples.

## 4. Discussion

Unclean water and food, unhygienic practices of caretakers and poor domestic hygiene (e.g., open defecation) have been identified as the three main risk causes of diarrhoea in children [34,35]. The proportion of toys that tested positive for total coliform and *E. coli* bacteria (Table 2, Table 3 and Table 4) in this study could be due to poor hygiene and sanitation aspects. In this study, children were reported to defecate in open spaces in the yard. Exposed faeces of children on the ground near homes or where children play increases the risk of transmitting faecal contaminants that could result in diarrhoea [36]. As a result, flies can act as mode of transmission and infect foods and contaminate toys and other fomites [35]. Lee et al. (2007) found that toys in DCCs were contaminated with bacterial pathogens which was also caused by poor sanitary behaviour [12]. Holaday et al. (1995) conducted a study in a DDCs in Tennessee (USA) and found that the toys were contaminated with total coliform and enteric bacteria which was associated with poor sanitary behaviour in diaper changing by staff [7]. Poor hygiene behaviour in children, especially in DCCs, has been shown to result in blood-borne infections and respiratory and gastrointestinal infections [5,8,11,15]. Another example of poor hygiene practice is that many people in the same space of time (usually during meals) wash their hands in the same water container and, depending on the availability of water, the water inside these washing trays was not replaced constantly over the duration of the day. This practice leads to people re-exposing themselves and the environment all the time to their own and other people’s bacteria [2]. 

Poor water quality has been shown to be a transmission route of coliform bacteria and several pathogenic *E. coli* strains [21]. People residing in rural areas of the Vhembe district rely mostly on untreated water sources for daily activities and it has been previously reported that several rural villages in the Vhembe district have poor water quality, and the water was found to be contaminated with high counts of coliforms [20,21]. In this study, people residing in rural communities reported to rely on open water sources because communal or yard taps does not provide water for periods up to 4 weeks. Obi and colleagues (2004) investigated different river sources in the Vhembe district and found that all the water sources were contaminated with several of the diarrhoeagenic *E. coli* strains [21]. These *E. coli* strains produce virulence factors that can be fatal, especially if these diarrhoeal pathogens can occur in multiple pathogen infections [23]. Human activities such as laundry and diaper disposal, and presence of human excreta and animal grazing have been observed in various rivers around the Vhembe region where water is being collected for drinking and stored in containers at the point-of-use under poor hygienic conditions [21]. Obi et al. (2004) found that people living in the Vhembe region had diarrhoea that was caused by EPEC and the water sources (rivers) were also contaminated with EPEC and EAEC [21]. As most of the rural households in the Vhembe region use water sources from rivers, boreholes and unprotected wells [20], the children can easily be exposed to these pathogens when drinking contaminated water or water that has been inadequately treated. A recent study done in the rural Vhembe region showed that children under the age of 5 years had diarrhoea caused by ETEC, EPEC, EAEC and other diarrhoeal pathogens [23]. In many cases, these virulent *E. coli* strains infect the children in combinations which make the infection more severe. In other studies, done in rural areas, combination of pathogenic *E. coli* was observed [23,37]. In Terhan, it was found that EHEC, EAEC, ETEC and EPEC combinations were more common in children less than one year of age [38]. In India, children had diarrhoea which was caused by combinations of EPEC, EIEC, ETEC, EAEC and EHEC strains [37]. Albert and colleagues (1995) found that children less than five years in Bangladesh had acute diarrhoea that was caused by EPEC and ETEC strains [39]. 

In this study, existing toys from DCCs were mostly infected with ETEC. ETEC is characterised by colonising the small intestine. After adherence, it produces either heat-stable (ST) or heat-liable (LT) toxin, resulting in acute watery diarrhoea [40]. In Indonesia, ETEC was found to cause diarrhoea in all age groups but was commonly found in young children [41]. EHEC produces the Shiga toxin which causes bloody diarrhoea and can be fatal in certain individuals [26]. Shiga toxin-producing *E. coli* causes bloody diarrhea, and causes potentially fatal diseases in humans, including hemolytic-uremic syndrome (HUS) and haemorrhagic colitis [26]. In this study, existing toys from households had high counts of EHEC compared to the introduced toys. Koyange et al. (2004) found that children in Kinshasa had bloody diarrhoea which was caused by EHEC [42]. Most cases of EIEC present with watery diarrhoea indistinguishable from STEC [43]. EPEC mainly causes infantile diarrhoea and is characterised by the presence (typical EPEC) or absence (atypical EPEC) of bundle-forming pili [44]. Typical EPEC mostly infects children under the age of 12 months with most cases resulting in death [45]. In this study, toys introduced to DCCs were found to have only aEPEC, while tEPEC was found across all groups, except in introduced DCC toys.

## 5. Conclusions

Hygiene plays an important role in the well-being of young children. Children, especially under the age of 5 years, are at risk of transmitting pathogens as they have not yet mastered good sanitation and hygiene behaviour. Rural settings such as the Vhembe district have a lack of access to improved water quality, sanitation and hygiene behaviour. Most people residing in these settings rely mostly on open water sources which are contaminated with various diarrhoeal pathogens due to human and animal activities [21]. Children are therefore easily exposed to these pathogens due to their developing immune system. In this study, children’s toys from both households and DCCs were contaminated with total coliform and pathogenic *E. coli* strains. The limitations of the study were the sample size collected and that during collection of samples, some of the households did not give back the introduced toys. Another limitation of this study was the withdrawal of two DCCs from the study just after it began—the reason being that they did not want to be exposed, even though the approved ethical forms were explained, and a copy was provided. Several diarrheagenic *E. coli* pathotypes were detected in both existing and introduced toys. It is therefore recommended that children’s toys are kept clean by washing toys regularly with soap in order to reduce transmission of pathogens [35]. WASH aspects do play a major role in the well-being of children and more risk assessment studies are needed to identify health risks in order to implement interventions. More educational messages should also be advocated to show mothers and caretakers of young children the importance of washing toys and sterilising fomites in households and DCCs. 

## Figures and Tables

**Table 1:** Primers used for detection of diarrhoeagenic *E. coli* strains (Omar and Barnard, 2014).

Pathogen	Primer	Sequence	Size (bp)	Reference
Commensal *E. coli*	*Mdh(F)* *Mdh (R)*	GGT ATG GAT CGT TCC GAC CT GGCAGA ATG GTA ACA CCA GAG T	304	[28]
EIEC	*ial (F)* *ial (R)*	GGT ATG ATG ATG AGT AGT CCA GGA GGC CAA CAA TTA TTT CC	650	[29]
EHEC/Atypical EPEC	*eaeA (F)* *eaeA (R)*	CTG AAC GGC GAT TACGCG AA CCA GAC GAT ACG ATC CAG	917	[30]
Typical EPEC	*bfpA (F)* *bfpA (R)*	AAT GGT GCT TGC GCT TGC TGC TAT TAA CAC CGT AGC CTT TCG CTG AAG TAC CT	410	[30]
EAEC	*stxl (F)* *stxl (R)* *stx2 (F)* *stx2 (R)*	ACA CTG GAT GAT CTC AGT GG CTG AAT CCC CCT CCA TTA TG CCA TGA CAA CGG ACA GCA GTT CCTGTC AAC TGA GCA CTT TG	614	[31]
ETEC	*lt (F)* *lt (R)*	GGC GAC AGA TTA TAC CGT GC CGG TCT CTA TAT TCC CTG TT	360	[32]
External control	*gapdh (F)* *gapdh (R)*	GAG TCA ACG GAT TTG GTC GT TTG ATT TTG GAG GGA TCT CG	238	[33]

**Table 2:** Descriptive sanitation demographics for the study households (HH) and DCCs.

Variable	HH (n = 54)	DCC (n = 6)
**A toilet facility was present at HH/DCC**	44 (81%)	6 (100%)
Type of toilets present *		
Flush toilet	4 (7%)	2 (33%)
Pit latrine	45 (83%)	4 (67%)
Bucket system	5 (9%)	-
**Alternate toilet for children**		
Open space in the yard	16 (30%)	3 (50%)
Open space in the bush	5 (9%)	-
Children’s toilet	19 (35%)	3 (50%)
No data	14 (26%)	-

* Some households had more than one type of sanitation facility present.

**Table 3:** Descriptive water demographics for the study households and DCCs.

Variable	HH (n = 54)	DCC (n = 6)
**Water source**		
Communal tap	22 (41%)	2 (33%)
Tank	8 (15%)	-
Borehole	3 (6%)	1 (17%)
River water	10 (19%)	-
Communal tap and borehole	4 (7%)	1 (17%)
Tank and river water	7 (13%)	2 (33%)
Is water sometimes not available *	54 (100%)	4 (67%)
How often is water not available at the source? **		
Weekly	10 (19%)	3 (50%)
Monthly	32 (59%)	1 (17%)
Annually	12 (22%)	-
Use alternative source for how long **		
Days	7 (13%)	-
Week	23 (43%)	2 (33%)
Month	11 (20%)	2 (67%)
No data	13 (24%)	-
Estimated distance to collect water **		
0–10 m	5 (9%)	-
10–50 m	9 (17%)	-
50–100 m	10 (19%)	3 (50%)
100–200 m	12 (22%)	1 (17%)
>200 m	18 (33%)	-
Transport of water to household/DCC **		
Carry on head	20 (37%)	1 (17%)
Wheelbarrow	31 (57%)	3 (50%)
No data	3 (6%)	-

** Answers, based on reply to Question * as indicated in the table.

**Table 4:** Descriptive hygiene demographics for the study households and DCCs.

Variable	HH (n = 54)	DCC (n = 6)
**Hand washing site**		
Washing facility close to toilet	12 (22%)	-
Dish washing container	15 (28%)	4 (67%)
Use drinking beaker to pour water to wash hands	19 (35%)	2 (33%)
At source such as river/tap	2 (4%)	-
At water storage containers	1 (2%)	-
No data	5 (9%)	-
**When do caretakers of the child wash their hands?**		
Visibly soiled	7 (13%)	3 (50%)
After touching something contaminated	30 (56%)	4 (67%)
After using the toilet	44 (81%)	5 (83%)
After changing nappies	37 (69%)	6 (100%)
Before preparing food	43 (80%)	6 (100%)
Before meals	43 (80%)	6 (100%)
**Food hygiene**		
Wash dishes with soap and warm water	54 (100%)	6 (100%)
Cover food with cloth/lid	45 (83%)	6 (100%)
No method	9 (17%)	-
Rinse food	54 (100%)	6 (100%)

**Table 5:** Frequency of total coliform and *E. coli* (counts/100 mL) on existing and introduced toys in rural households (HHs) and day care centres (DCCs) *.

		WHO Criteria (WHO, 2001)			
		0 cfu/100 mL	1–10 cfu/100 mL	10–100 cfu/100 mL	>100 cfu/100 mL
**Households**	**Total coliform Existing toys**	6% (3/54)	13% (7/54)	19% (10/54)	63% (34/54)
	**Total coliform Introduced toys**	4% (2/54)	9% (5/54)	19% (10/54)	69% (37/54)
	***E. coli*** **Existing toys**	63% (34/54)	13% (7/54)	20% (11/54)	4% (2/54)
	***E. coli*** **Introduced toys**	67% (36/54)	11% (6/54)	6% (3/54)	17% (9/54)
**DCCs**	**Total coliform Existing toys**	7% (4/55)	31% (17/55)	33% (18/55)	29% (16/55)
	**Total coliform Introduced toys**	4% (2/55)	24% (13/55)	36% (20/55)	36% (20/55)
	***E. coli*** **Existing toys**	78% (43/55)	20% (11/55)	2% (1/55)	0% (0/55)
	***E. coli*** **Introduced toys**	86% (47/55)	9% (5/55)	4% (2/55)	2% (1/55)

* Excluding data from 2 DCCs who withdrew from study.

**Table 6:** Geometric mean (cfu/100 mL) and 95% CI for total coliform and *E. coli* data from toys *.

Household toys				DCC toys			
Total Coliform		*E. coli*		Total Coliform		*E. coli*	
Existing [95% CI]	Introduced [95% CI]	Existing [95% CI]	Introduced [95% CI]	Existing [95% CI]	Introduced [95% CI]	Existing [95% CI]	Introduced [95% CI]
278 [135; 572]	344 [185; 639]	14 [5; 35]	100 [21; 467]	27 [16; 50]	55 [30; 103]	24 [14; 41]	52 [29; 95]

* Excluding data from 2 DCCs who withdrew from study.
